# Selective suppression of de novo SARS-CoV-2 vaccine antibody responses in patients with cancer on B cell–targeted therapy

**DOI:** 10.1172/jci.insight.163434

**Published:** 2023-03-22

**Authors:** Joseph H. Azar, John P. Evans, Madison H. Sikorski, Karthik B. Chakravarthy, Selah McKenney, Ian Carmody, Cong Zeng, Rachael Teodorescu, No-Joon Song, Jamie L. Hamon, Donna Bucci, Maria Velegraki, Chelsea Bolyard, Kevin P. Weller, Sarah A. Reisinger, Seema A. Bhat, Kami J. Maddocks, Nathan Denlinger, Narendranath Epperla, Richard J. Gumina, Anastasia N. Vlasova, Eugene M. Oltz, Linda J. Saif, Dongjun Chung, Jennifer A. Woyach, Peter G. Shields, Shan-Lu Liu, Zihai Li, Mark P. Rubinstein

**Affiliations:** 1 Division of Medical Oncology, Department of Internal Medicine;; 2 The Pelotonia Institute for Immuno-Oncology, The Ohio State University Comprehensive Cancer Center – James;; 3 Center for Retrovirus Research;; 4 Department of Veterinary Biosciences;; 5 Molecular, Cellular and Developmental Biology Program;; 6 The Ohio State University Comprehensive Cancer Center – James, The James Cancer Hospital;; 7 Division of Hematology, Department of Internal Medicine, The Ohio State University Comprehensive Cancer Center – James;; 8 Department of Internal Medicine, Division of Cardiovascular Medicine; and; 9 Center for Food Animal Health, Animal Sciences Department, Ohio Agricultural Research and Development Center, College of Food, Agricultural and Environmental Sciences, The Ohio State University, Columbus, Ohio, USA.; 10 Veterinary Preventive Medicine Department, College of Veterinary Medicine, The Ohio State University, Wooster, Ohio, USA.; 11 Viruses and Emerging Pathogens Program, Infectious Diseases Institute;; 12 Department of Microbial Infection and Immunity; and; 13 Department of Biomedical Informatics, The Ohio State University, Columbus, Ohio, USA.

**Keywords:** COVID-19, Oncology, Cancer, Immunoglobulins

## Abstract

We assessed vaccine-induced antibody responses to the SARS-CoV-2 ancestral virus and Omicron variant before and after booster immunization in 57 patients with B cell malignancies. Over one-third of vaccinated patients at the pre-booster time point were seronegative, and these patients were predominantly on active cancer therapies such as anti-CD20 monoclonal antibody. While booster immunization was able to induce detectable antibodies in a small fraction of seronegative patients, the overall booster benefit was disproportionately evident in patients already seropositive and not receiving active therapy. While ancestral virus– and Omicron variant–reactive antibody levels among individual patients were largely concordant, neutralizing antibodies against Omicron tended to be reduced. Interestingly, in all patients, including those unable to generate detectable antibodies against SARS-CoV-2 spike, we observed comparable levels of EBV- and influenza-reactive antibodies, demonstrating that B cell–targeting therapies primarily impair de novo but not preexisting antibody levels. These findings support rationale for vaccination before cancer treatment.

## Graphical abstract

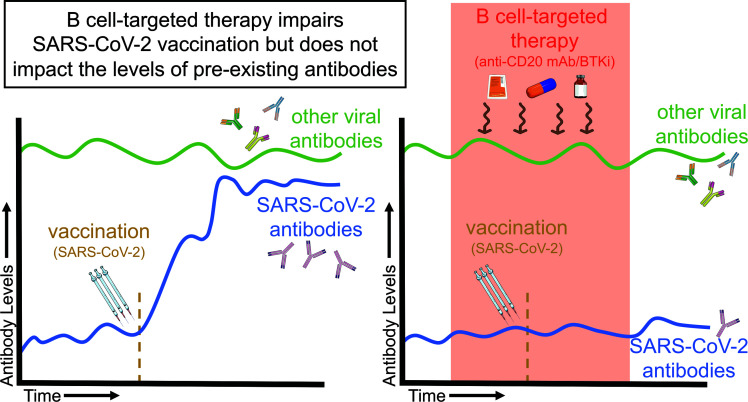

## Introduction

The Omicron (B.1.1.529) SARS-CoV-2 variant exhibits over 30 mutations in the spike protein, and 15 mutations in the receptor-binding domain (RBD), relative to the ancestral strain (1–3). These mutations may allow evasion of SARS-CoV-2 immunity, particularly antibody responses, and therefore overcome vaccine-mediated immunity in some individuals (2, 4, 5). While emerging data suggest that in healthy immunized individuals there is sufficient immune protection against serious disease (6), the level of vaccine-induced protection in immunocompromised individuals such as patients with cancer is not well known. This is of particular importance to patients with hematological malignancies, who have a higher risk of severe disease or death from COVID-19 than solid-tumor patients (7–9), and for whom emerging data suggest continued elevated risk from COVID-19 even after vaccination (9–14). A third dose of the mRNA vaccine (booster) can augment antibody responses in cancer and organ transplant patients who previously received a primary (2-dose) mRNA vaccine (15–18). We and others have also reported that in healthy individuals and solid-tumor patients, booster immunization can substantively augment neutralizing antibodies including against the Omicron variant (2–4, 15, 19–23). However, comparatively little is known about the impact of booster immunization in highly immunocompromised patients such as those with B cell malignancies, particularly those patients on active therapy. Such immune impairment may be particularly problematic for patients with chronic lymphocytic leukemia (CLL) or non-Hodgkin’s lymphoma, in whom B cell–targeted therapies have been shown to impair primary vaccine-mediated antibody responses (16, 23–29). Recent studies suggest that booster immunization is less effective in patients with hematological malignancies, including the induction of Omicron-neutralizing antibodies (16, 23, 29–31). However, these studies provided only a limited assessment of the impact of booster immunization and active B cell–targeted therapy on antibody levels. Here, we evaluate the impact of booster immunization on levels of antibodies specific for Omicron and ancestral variants in cancer patients with or without active B cell–targeted therapy. Furthermore, we assess the extent to which B cell–targeted therapies suppress de novo SARS-CoV-2 antibody levels versus preexisting antibody levels against endemic viruses, which has not been previously assessed in patients remaining seronegative after vaccination.

## Results

### Patients and therapies received.

Our goal was to assess mRNA vaccine (mRNA-1273 or BNT162b2)–induced antibody responses to Omicron (B.1.1.529) in comparison with ancestral SARS-CoV-2 in a highly immunocompromised patient population. Accordingly, we evaluated 57 patients with CLL (*n* = 35) and non-Hodgkin’s lymphoma (*n* = 22) who had paired serum samples before and after booster immunization (Table 1). Thirty of the 57 patients were on active therapy, including 6 patients on anti-CD20 or anti-BAFF monoclonal antibody (mAb), 15 patients on Bruton tyrosine kinase (BTK) inhibitors, and 7 patients receiving the combination of these agents. Six of the 30 patients stopped active therapy before booster immunization, with drug stoppage being in a range of 4 months before to 4 months after primary vaccination. Of the remaining 27 patients without active therapy, 9 were untreated for their disease, and the remaining 18 had received prior B cell–targeting therapies (mean = 4.9 years, range 15 months to 15 years before primary vaccination). The first paired sample was collected 66–216 days (mean 139 days) after the primary vaccination (second mRNA vaccine dose), and the second paired sample was collected 5–112 days (mean 52 days) after the booster vaccination. The time range between first and second sample collection was 21–182 days (mean 94 days). Details related to patient demographics are included in Table 1.

### Compromised antibody levels in boosted cancer patients undergoing B cell–targeted therapy.

**Figure 1:**
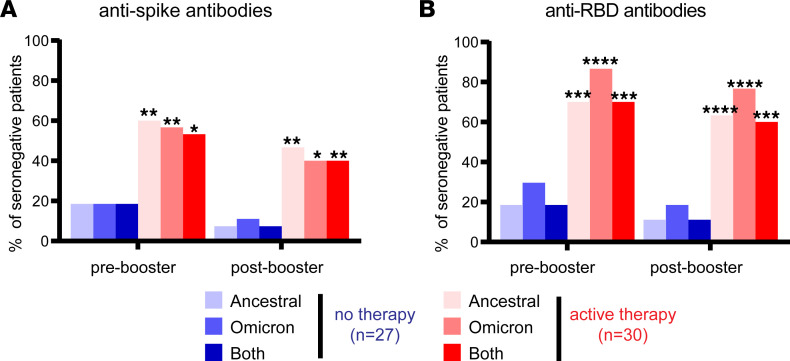
Percentages of patients without antibodies against the spike or RBD protein of ancestral SARS-CoV-2, Omicron variant, or both. (**A**) Each bar depicts the percentage of patients deficient in detectable antibodies against the ancestral spike or Omicron spike or antibodies recognizing either target as determined by ELISA. (**B**) Percentages of patients deficient in detectable antibodies against ancestral RBD or Omicron RBD or antibodies recognizing either target. Fisher’s exact test was conducted to compare the number of seronegative patients with no active therapy and active therapy. Shown are comparisons between active therapy and no therapy for the same antibody evaluation and group. *P < 0.05, **P < 0.01, ***P < 0.001, ****P < 0.0001. Fisher’s exact test was also conducted to compare ancestral, Omicron, or both within individual groups, and the differences were not statistically significant.

**Figure 2:**
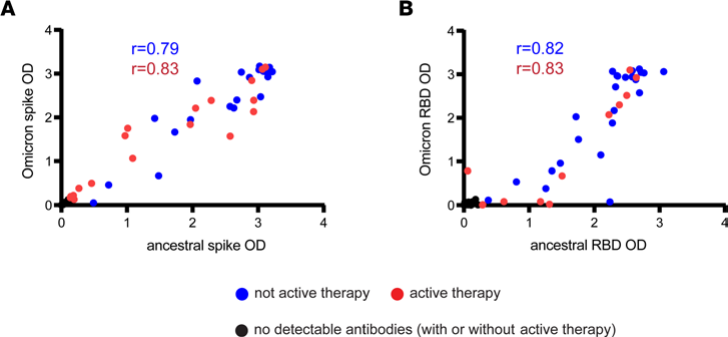
Positive correlation between antibody levels against ancestral and Omicron spike and RBD post-booster. (**A**) Dot plot shows the post-booster patient time points graphed by Omicron spike versus ancestral spike as determined by ELISA. Red dots depict patients on active therapy, blue dots depict patients not on active therapy, and black dots indicate patients without any detectable antibodies. (**B**) Same as **A** except showing Omicron RBD versus ancestral RBD. The Spearman’s rank correlation coefficient was calculated for patients on active therapy (red) or not on active therapy (blue) with exclusion of patients without detectable antibodies.

As a first step in understanding how targeted B cell therapies impact SARS-CoV-2 humoral responses following vaccination and/or boosting, we determined the proportion of patients who were seronegative for antibodies reactive to ancestral and Omicron spike protein using an enzyme-linked immunosorbent assay (ELISA). After primary immunization but before booster administration, we found that the proportion of patients seronegative for both ancestral and Omicron spike was significantly elevated in those who received active therapy versus those not receiving active therapy (53% [16 of 30 patients] vs. 18% [5 of 27 patients], *P* < 0.05) (Figure 1). Upon booster immunization, the lack of detectable antibodies continued to be disproportionate in patients with active therapy versus those not receiving active therapy (40% [12 of 30 patients] vs. 7.4% [2 of 27], *P* < 0.01) (Figure 1A). Similar results were obtained using ELISA to detect antibodies reactive against ancestral and Omicron RBD (Figure 1B). Of patients who were seronegative for either ancestral or Omicron spike before booster administration, only 33% (7 of 21) converted to seropositivity after booster administration. Similarly, 19% (5 of 26) of patients without detectable antibodies against either ancestral or Omicron RBD seroconverted after booster administration. Of the 6 patients who stopped active therapy before booster administration, all were seronegative before and after booster administration, and all had been treated with anti-CD20 or anti-BAFF mAb. Importantly, upon analysis of patients with detectable antibodies against spike or RBD, there was a positive correlation between antibody levels against the ancestral and Omicron spike and RBD independent of treatment (Figure 2). For example, there was a positive correlation after booster administration between ancestral RBD and Omicron RBD in patients with (*r* = 0.83) or without (*r* = 0.82) active therapy. Collectively, these results demonstrate a severe deficiency in the ability to develop vaccine-associated antibodies in cancer patients with active B cell–targeted therapies but highlight that patients with detectable antibodies exhibit substantial overlap in reactivity between the ancestral and Omicron SARS-CoV-2 variant.

**Figure 3:**
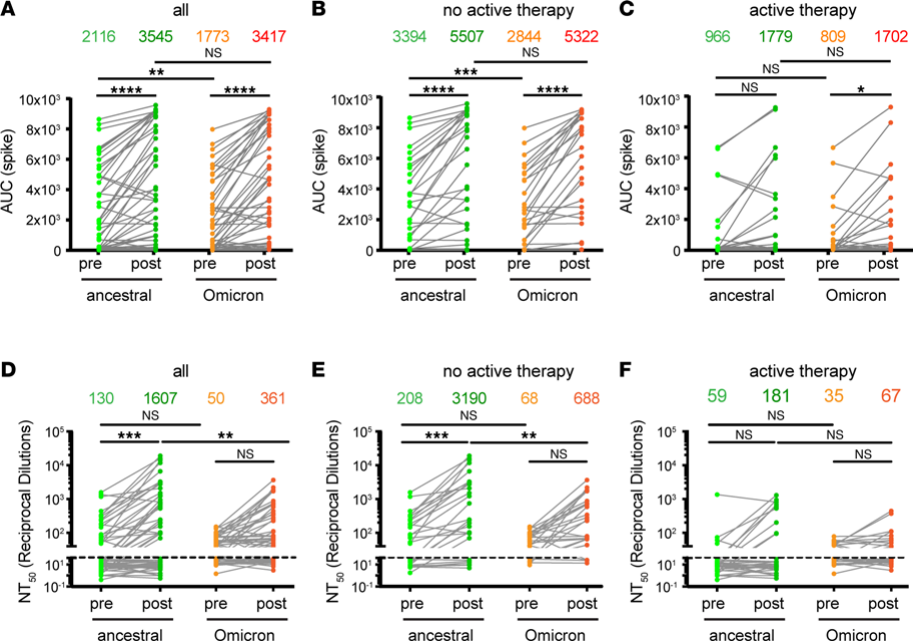
ELISA and viral neutralization assay depict variable antibody response to the ancestral and Omicron SARS-CoV-2 strains before and after booster vaccination in patients with hematological cancer. (**A**–**C**) Area under the curve (AUC) for all patients (**A**), patients without active therapy (**B**), and patients with active therapy (**C**). (**D**–**F**) Same as **A**–**C** except showing the 50% neutralization titer (NT_50_). For all data, each symbol represents a time point at which serum was isolated, and the line connects individual patients. The number above each graph represent arithmetic mean. ANOVA was used to compare between pre-booster ancestral, post-booster ancestral, pre-booster Omicron, and post-booster Omicron NT_50_/AUC values of the same patient. *P < 0.05, **P < 0.01, ***P < 0.001, ****P < 0.0001.

We next assessed relative levels of antibodies against ancestral and Omicron spike across all patients (Figure 3, A–C). While booster immunization clearly enhanced both ancestral virus–reactive and Omicron variant–reactive spike antibody levels in patients without active therapy (3,394 vs. 5,507, *P* < 0.0001, for ancestral spike; and 2,844 vs. 5,322, *P* < 0.0001, for Omicron spike) (Figure 3B), results for patients with active therapy were less clear-cut. Although there was a trend for increased levels of ancestral virus–reactive spike antibodies following booster administration in patients with active therapy, this trend was not statistically significant. However, for Omicron variant–reactive spike antibodies, the post-booster increase was significant (809 vs. 1,702, *P* < 0.05) (Figure 3C). Notably, for patients on active therapy, 23% (7 of 30) showed clear increases in antibodies against both ancestral and Omicron spike, suggesting that a subset of patients benefited from the booster. The impact of the booster was similar on ancestral virus–reactive and Omicron variant–reactive RBD antibodies (Supplemental Figure 1; supplemental material available online with this article; https://doi.org/10.1172/jci.insight.163434DS1). In addition to the deficiency in booster-mediated immunity apparent in patients on active therapy, these ELISA results support previous findings that patients with active therapy are deficient in generating antibodies after primary vaccination (16, 23, 24, 27–29). Thus, pre-booster antibody levels in patients with active therapy were significantly reduced for all targets evaluated by ELISA, including ancestral spike (*P* < 0.001), Omicron spike (*P* < 0.001), ancestral RBD (*P* < 0.001), and Omicron RBD (*P* < 0.01) (Supplemental Figure 1). For an illustrative comparison of antibody responses between patients, we subdivided patients into 4 groups based on their antibody response to ancestral RBD, including patients with (a) good responses to both primary and booster vaccine, (b) good responses to primary vaccine only, (c) good responses to booster vaccine only, and (d) poor responses overall (Supplemental Figure 2A). We observed similar patterns of antibody levels before and after booster against ancestral RBD, Omicron RBD, ancestral spike, and Omicron spike, again suggesting the induction of broadly cross-reactive antibodies. We also evaluated IgA and IgM antibody responses against the ancestral Spike protein (Supplemental Figure 2B). While we observed post-booster increased levels of spike-reactive IgA (*P* < 0.001) and IgM (*P* < 0.001) in individuals without active therapy, there was not a statistically significant increase in these antibodies for patients undergoing active treatment. Notably, IgG was the dominant antibody detected, and we did not observe patients with IgA or IgM in the absence of IgG (Supplemental Figure 2B). Finally, as a comparison, Supplemental Figure 1I shows antibody levels against ancestral and Omicron spike in healthy individuals compared with patients with cancer.

### Booster-augmented neutralizing antibodies against the SARS-CoV-2 ancestral and Omicron variant are increased in patients with B cell malignancy without active therapy.

To determine whether antibody levels correlated with functional blockade of SARS-CoV-2 pseudovirus, we assessed neutralizing antibody response to the ancestral and Omicron variants of SARS-CoV-2 using our previously reported pseudotyped lentivirus neutralization assay (15, 32). In these experiments, we used ancestral D614G as a reference for comparison, then determined the change in 50% neutralization titer (NT_50_) before and after booster in all patients (Figure 3D). For the ancestral strain, there was a 12-fold increase in NT_50_ (from 130 to 1,607; *P* < 0.001) before and after booster immunization. In contrast, for the Omicron variant, there was only a 7-fold increase in NT_50_ (from 50 to 361); the latter increase was not significant, possibly because 39% (22/57) of patients had an NT_50_ below the level of detection against Omicron following booster vaccine administration. Notably, NT_50_ titers against the Omicron strain post-booster exhibited a 4.5-fold reduction (1,607 vs. 361, *P* < 0.001) in comparison with the ancestral strain, consistent with previous findings (2–4, 15, 19, 20, 23).

In keeping with our ELISA results, patients not on active therapy had substantially higher titers of neutralizing antibodies after booster administration (Figure 3, E and F). Thus, for patients without active therapy, there was a 15-fold increase in NT_50_ (208 to 3,190, *P* < 0.001) in ancestral neutralizing antibodies (Figure 3E). In comparison, after booster administration, there was an 11.5-fold increase in NT_50_ in Omicron-neutralizing antibodies (68 to 688), although this difference did not attain statistical significance. In patients with active B cell–targeting therapies, there was a minimal increase in ancestral neutralizing or Omicron-neutralizing antibodies, and these differences were not significant. However, there appeared to be a small subset of active-treatment patients who benefited from the booster (Figure 3F). Overall, these results demonstrate that patients on active B cell–targeted therapies exhibited substantively diminished booster responses, as measured by induction of neutralizing antibodies. Furthermore, SARS-CoV-2 mRNA vaccination using the ancestral spike protein was able to induce neutralizing antibodies against Omicron in many patients, though predominantly in patients without active therapy.

### Clinical correlates and monoclonal antibody therapy.

In-depth analysis of clinical parameters associated with the detection of antibodies is provided in Supplemental Table 1. Likely owing to insufficient patient numbers, we were unable to draw significant conclusions as to the impact of disease (CLL and non-Hodgkin’s lymphoma) or specific therapy, though these parameters are illustrated graphically (Supplemental Figure 1). There was also not a clear association between clinical parameters such as type of treatment, sex, age, days after booster, white blood cell counts at booster, and absolute lymphocyte counts at booster. Graphical display of longitudinal analysis of all study patients plotted versus their booster time point illustrated the impact of active therapy on antibody levels (Supplemental Figure 3).

As an illustrative example of detection of therapeutically administered mAb, we assessed additional post-booster samples taken from 3 patients who were initially seronegative after primary vaccination. These patients tested positive for infection with SARS-CoV-2 and, within 4 days, were treated with Regeneron mAb cocktail (casirivimab and imdevimab). Full details are provided in Supplemental Figure 4. In these patients, we detected remarkable antibody selectivity for ancestral RBD in comparison with Omicron RBD, consistent with detection of the Regeneron mAb cocktail, which has been reported to not efficiently recognize Omicron RBD (1–3, 33). These results are consistent with detection of infused antibodies and lack of endogenous antibody responses despite booster and infection. Our results are noteworthy as many immunosuppressed patients are likely to be treated with mAb therapy, which may be impactful on antibody correlative studies when full patient clinical data may not be available.

### B cell–targeted therapy selectively impacts de novo versus preexisting antibody levels.

**Figure 4:**
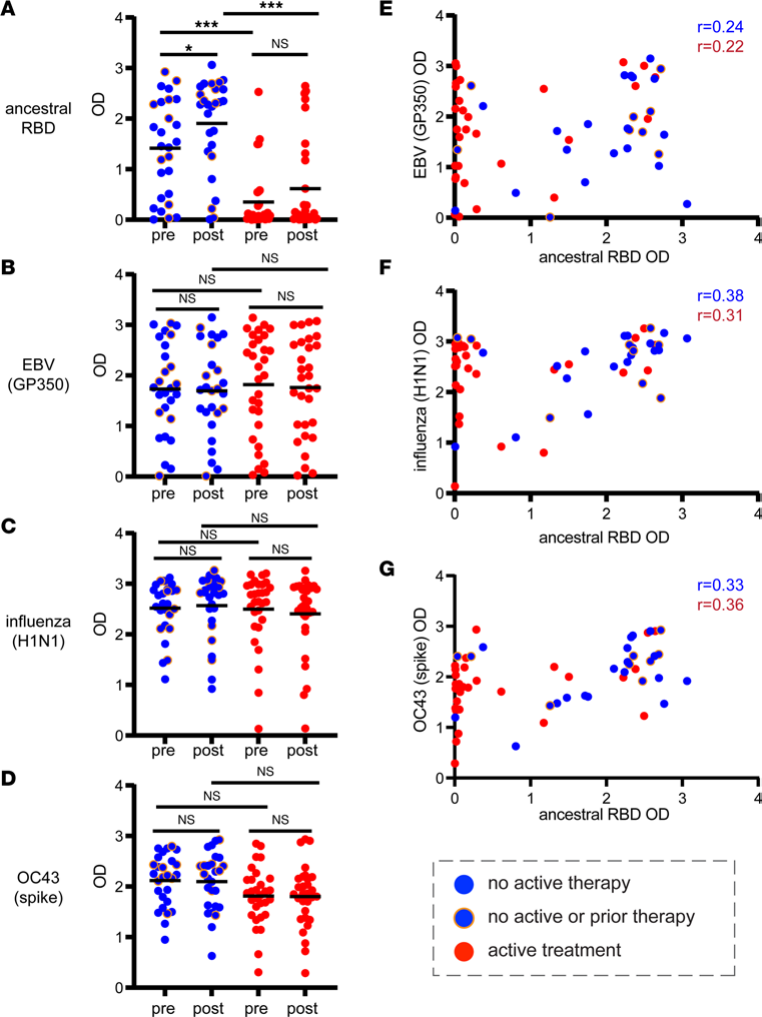
B cell–depleting therapy selectively impairs de novo antibody generation. (**A**) Antibody levels determined by ELISA against the ancestral RBD protein. Each patient is represented by a dot pre- and post-therapy. Patients without active therapy are indicated in blue, while patients on active therapy are indicated in red. Orange circles around blue dots indicate patients who are untreated. The arithmetic mean is shown by the bar. (**B**) Same as **A** except showing antibodies against the EBV GP350. (**C**) Same as **A** except showing antibodies against influenza H1N1. (**D**) Same as **A** except showing antibodies against the OC43 common cold coronavirus spike protein. (**E**) Dot plot of ancestral RBD versus EBV GP350 antibody binding taken from post-booster data. (**F**) Same as **E** except showing ancestral RBD versus influenza H1N1. (**G**) Same as **E** except showing ancestral RBD versus OC43 spike. For **A**–**D**, Wilcoxon’s signed-rank test was used to compare OD values between pre- and post-booster, while Mann-Whitney test was used to compare between different conditions (treated and nontreated), and the Bonferroni correction was applied. *P < 0.05, ***P < 0.001. For **E**–**G**, the Spearman’s rank correlation coefficient was calculated.

An important question is whether B cell–targeted therapies impair the maintenance of preexisting antibody levels as well as de novo antibody generation. To assess this question, we measured relative levels of antibodies against (a) EBV (GP350), (b) influenza, H1N1, A/Brisbane/2018 (HA protein), and (c) the common cold coronavirus OC43 (spike), in addition to the ancestral RBD (Figure 4, A–D). In contrast to antibodies against SARS-CoV-2 RBD, those specific for EBV, influenza, and the common coronavirus were not significantly different in patients with active treatment versus no active treatment. Importantly, there was no correlation between lack of ancestral RBD antibodies and lack of antibodies against any of the other viral targets (Figure 4, E–G). More extensive analysis is provided in Supplemental Figure 5, including results obtained for other viral targets, and patients subdivided into groups with different levels of vaccine response against the ancestral RBD protein. These results demonstrate that preexisting antibody levels against endemic viruses were not altered even in patients without detectable antibodies against ancestral RBD following SARS-CoV-2 vaccination and booster.

## Discussion

We observed substantial heterogeneity in antibody responses following booster immunization in patients with B cell malignancies. While patients not on active cancer therapy showed significant increases in antibody levels after booster immunization, many patients on active therapy remained seronegative despite primary immunization and booster. Importantly, among all seropositive patients, there was a strong correlation between antibodies against the ancestral strain and Omicron variant. These findings indicate that Omicron is not sufficiently antigenically distinct to escape all vaccine-mediated antibody immunity. However, as previously reported in healthy individuals and solid-tumor patients (2–4, 15, 16, 19, 20, 23), we found in B cell malignancy patients that neutralizing antibody levels against Omicron were also reduced in comparison with the ancestral virus. In addition to providing new insights into Omicron-related immunity, our results build on existing data for ancestral strain immunity showing that B cell–targeted therapies are associated with reduced antibody responses after primary vaccination (24, 27–29, 34, 35) and that a significant fraction of patients with B cell malignancies remain seronegative after booster immunization (16, 23, 29, 30, 36). These findings contrast with studies in solid-tumor patients, in which the majority of patients develop robust booster-mediated responses, though cytoreductive chemotherapies may impair responses in a subset of solid-tumor patients (15, 21, 24, 27, 37). While our current study has focused on antibody levels, antigen-specific B and T cells are likely also critical for protective immunity. Notably, memory B cell levels can increase while antibody levels decrease (38). In multiple sclerosis patients receiving anti-CD20 mAb, there were elevated SARS-CoV-2–reactive CD8^+^ T cell responses (35), suggesting compensatory immune pathways. Recent findings also show, as with antibody responses, T cell cross-reactivity between the ancestral and Omicron variants (39, 40). Together, our results demonstrate that booster immunization may be beneficial to select patients with B cell malignancies; however, further studies will be needed to determine whether post-booster seronegative patients may also benefit from their vaccination regimen.

A second important finding in our study was that regardless of whether patients were on active therapy or had an antibody response to primary or booster SARS-CoV-2 vaccination, patients maintained near-normal levels of preexisting antibodies against endemic viruses including influenza and EBV. While many studies in humans in the pre–SARS-CoV-2 era showed that B cell–targeted therapies impair vaccine-mediated antibody responses (41–46), these studies did not assess preexisting antibody levels such as those that might provide protection against endemic viruses. Thus, in patients with B cell–targeted therapy in whom vaccination fails to generate detectable antibodies, are preexisting antibody levels compromised? While this question has not been previously assessed, a handful of studies have assessed preexisting antibody levels in the context of B cell–depleting therapy. In mice and macaques, preexisting antibodies, including those generated following vaccination and infection, were preserved after anti-CD20 mAb treatment (47, 48). In rheumatoid arthritis patients treated with anti-CD20 mAb, levels of antibodies against tetanus toxoid and pneumococcal capsular polysaccharides, and also autoantibodies, appeared to be maintained (49, 50). Similar findings were also suggested in multiple sclerosis patients treated with anti-CD20 mAb, although limited data were provided (41, 51). Our data build on these observations and, importantly, further demonstrate that in patients who remain completely seronegative following vaccination, preexisting antibodies are maintained. Our findings apply to patients treated with anti-CD20 mAb as well as BTK inhibitors. Our results indicate that the principles regulating de novo antibody generation and maintenance of preexisting antibodies are fundamentally distinct. Our findings suggest that B cell–targeted therapies have minimal impact on long-lived plasma cells (LLPCs), which are likely the source of basal and preexisting antibody levels (52, 53). The ability of LLPCs to avoid anti-CD20 mAb and BTK inhibitor therapy may reflect lack of CD20 expression, tissue sequestration from drug, and signal-independent expression of antibodies. While our findings suggest that B cell–targeted therapies do not impair the maintenance of preexisting antibody levels, future studies will be needed to assess protective immunity. However, our data suggest that vaccination prior to B cell–targeted therapy may be important to achieve long-term benefits. In support of this notion, 2 recent studies, with a relatively small number of patients, provide evidence that SARS-CoV-2 vaccination prior to anti-CD20 mAb infusion had superior induction of antibody responses compared with when the agents were administered in reverse order (54, 55).

In summary, our findings highlight the discordance between de novo and preexisting antibodies. Our findings provide a framework for further studies to understand antibody biology and to develop more effective vaccine strategies in patients with B cell–targeted therapies. Finally, our work suggests that patients with B cell malignancies on active therapy may be disproportionately vulnerable to emerging or new infections because of an inability to generate de novo antibody responses.

An important limitation in our study is that while active therapy is associated with blunted antibody responses, we cannot formally determine whether reduced antibody responses are due to therapy or more advanced disease. Other limitations of our study include a relatively low patient number, the inherent heterogeneity in patients in terms of both disease and treatment, and the variable range in time between vaccination and blood draws. While we excluded patients with SARS-CoV-2 therapeutic mAb treatment within 6 months of the post-booster blood draw, as our patient population is highly vulnerable, medical records may not have reflected mAb therapy performed at other sites. We also did not assess other immune correlates that may be impactful for protection including memory B cells, the presence of which may be more critical than antibody levels. Finally, while our data suggest importance of vaccination prior to initiation of B cell–targeted therapy, clinical studies will be necessary to validate this conclusion.

## Methods

### Human participants.

All cancer patients received 2 mRNA vaccine doses and 87–276 days later received an additional (third) mRNA booster dose. Cancer patient sera (*n* = 57) were collected before and after booster mRNA vaccine. Pre-booster samples were collected between 66 and 216 days after second mRNA vaccine, and post-booster samples were collected between 5 and 112 days after mRNA booster. All patients with cancer had diagnoses of B cell malignancies including CLL (*n* = 35) or non-Hodgkin’s lymphoma (*n* = 22). Patients were separated into those not on active therapy (*n* = 27) and those on active therapy (*n* = 30) based on B cell–targeted therapy being administered within 9 months of the primary vaccination. Two patients were included in the study who received mAb therapy within 3 months before primary vaccination. Clinical information was extracted from the internal electronic medical record database, including age, sex, race, cancer diagnosis and therapy status, COVID-19 mRNA vaccine information, clinical laboratory findings, and diagnosis/treatment of COVID-19 infection. All patient demographic and clinical data are further described in Table 1. One patient removed from the main cohort but reported in Supplemental Figure 4 was excluded because of administration of COVID-19 mAb shortly prior to post-booster sample collection (~8 days).

### ELISAs.

ELISAs were conducted as previously described (56). Briefly, the wells of 96-well plates (Costar) were coated with 2 μg/mL of target protein in PBS overnight at 4°C. Target proteins included SARS-CoV-2 proteins (ancestral spike, LakePharma; ancestral RBD, LakePharma; Omicron spike, Sinobiological; Omicron RBD, Sinobiological; EBV [GP350], Sinobiological; influenza A, H1N1, Brisbane/2018 [HA protein], Sinobiological; influenza A, H3N2, Cambodia/2020 [HA protein], Sinobiological; OC43 [spike], Sinobiological; HKU1 [spike], Sinobiological; NL63 [spike], Sinobiological; and 229E [spike], Sinobiological). The next day, the protein-coating solution was removed, and the plates were washed 3 times with PBS with 0.1% Tween-20 (PBS-T) using the Elx405 automated plate washer (Biotek). Plates were blocked with PBS-T solution with 3% dry powder milk and incubated at room temperature for 2 hours. Heat-inactivated (56°C for 60 minutes) serum samples were prepared the same day with dilutions in PBS-T and 1% milk powder solution, dispensed into the plate, and incubated at room temperature for 2 hours. Next, the plates were washed 3 times with 0.01% PBS-T, and an HRP-conjugated anti-human IgG, IgA, or IgM (goat anti-human IgG/IgA/IgM Fc cross-absorbed secondary antibody HRP, Invitrogen [catalog 31413, PA1-74395, 31415], used at 1:3,000 dilution) antibody solution in 1% milk powder in PBS-T solution was added to each well, followed by incubation in the dark at room temperature for 1 hour. After incubation, the solution was removed and the plates were washed 3 more times with 0.1% PBS-T. Immediately after addition of 1 mL of 10× stable peroxide substrate (Thermo Fisher Scientific) to a solution of one *o*-phenylenediamine dihydrochloride tablet (Thermo Fisher Scientific) in 9 mL of deionized water, 100 μL of this solution was dispensed into each well. After 10 minutes, 50 μL of 2.5 M sulfuric acid solution (Thermo Fisher Scientific) was added to each well to stop the reaction. The optical density (OD) values for the plates were read at 490 nm using the SpectraMax iD5 Multi-Mode Microplate Reader (Molecular Devices). ELISAs for the SARS-CoV-2 target proteins (spike, RBD, Omicron spike, Omicron RBD) were performed in 1 run per target protein with 3 dilutions (1:200, 1:800, and 1:3,200). Controls were assigned to every plate to account for interplate variability. The diluents’ average OD was subtracted to obtain the final OD values of the samples. Area under the curve was calculated using GraphPad Prism 9. ELISAs for other targets were performed at 1:200, and each target was assessed in 2 independent assays with similar results. For determination of detectable antibody levels, the cutoff was determined as 3 standard deviations above the mean of the negative control samples.

### Neutralization assay.

Pseudotyped virus neutralization assays were performed as previously described (15, 24, 32). Briefly, patient serum was 4-fold serially diluted and incubated with equivalent amounts of infectious D614G or Omicron (B.1.1.529) pseudotyped virus (final dilutions of 1:40, 1:160, 1:640, 1:2,560, 1:10,240, and no serum control). Following 1-hour incubation at 37°C, virus was used to infect HEK293T-ACE2 cells (NR-52511, BEI Resources Repository). *Gaussia* luciferase activity was assayed 48 hours and 72 hours after infection by combining of 20 μL of cell culture media with 20 μL of *Gaussia* luciferase substrate (0.1 M Tris pH 7.4, 0.3 M sodium ascorbate, 10 μM coelenterazine). Luminescence was measured by a BioTek Cytation5 plate reader. Neutralization curves were plotted in GraphPad Prism 5 using least-squares fit nonlinear regression to determine 50% neutralizing titers.

### Statistics.

GraphPad Prism 5 and 9 and R 4.1.1 were used for statistical analyses. Wilcoxon’s signed-rank test was used to compare paired values, and Mann-Whitney test was used to compare unpaired values. Multiple testing was adjusted using the Bonferroni correction. One-way ANOVA was used for multiple-group comparisons. For correlation studies, Spearman’s rank correlation was used. Fisher’s exact test was used for association analysis between 2 categorical variables. The significance level of 0.05 was used to determine significance; *P < 0.05, **P < 0.01, ***P < 0.001, and ****P < 0.0001. Statistical details for each graph or table are provided in the legends.

### Study approval.

Serum samples were collected from cancer patients enrolled under an approved IRB protocol (2021C004) as part of the SIIREN study (the COVID-19 Vaccine Study of Infections and Immune Response) at The Ohio State University Comprehensive Cancer Center. Written informed consent was received prior to participation.

## Author contributions

JHA, JPE, MHS, SM, IC, CZ, RT, DC, JAW, PGS, SLL, ZL, and MPR designed the research studies. Experiments were conducted by JHA, JPE, MHS, KBC, SM, IC, CZ, and RT. JHA, JPE, MHS, KBC, DC, SLL, ZL, and MPR led the analysis of data. JHA, JPE, MHS, KBC, DC, SLL, ZL, and MPR were responsible for curation of the data. JHA, JPE, MHS, and MPR wrote the original draft of the manuscript, and JHA, JPE, MHS, KBC, SM, IC, CZ, RT, NJS, JLH, DB, MV, CB, KPW, SAR, SAB, KJM, ND, NE, RJG, ANV, EMO, LJS, DC, JAW, PGS, SLL, ZL, and MPR contributed to editing and revising. JAW, PGS, SLL, and MPR supervised this effort, and KBC, SR, JAW, PGS, SLL, ZL, and MPR were responsible for project administration. JAW, PGS, SLL, ZL, and MPR conceptualized the study. JHA, JPE, MHS, and KBC share the first author position reflecting substantive contributions by all 4 individuals with regard to key aspects of the study and as outlined by their contributions listed above. While all 4 first coauthors contributed critical aspects of the manuscript, each contributed in different ways to the study, and the order reflects the importance of these contributions.

## Supplementary Material

Supplemental data

## Figures and Tables

**Table 1:**
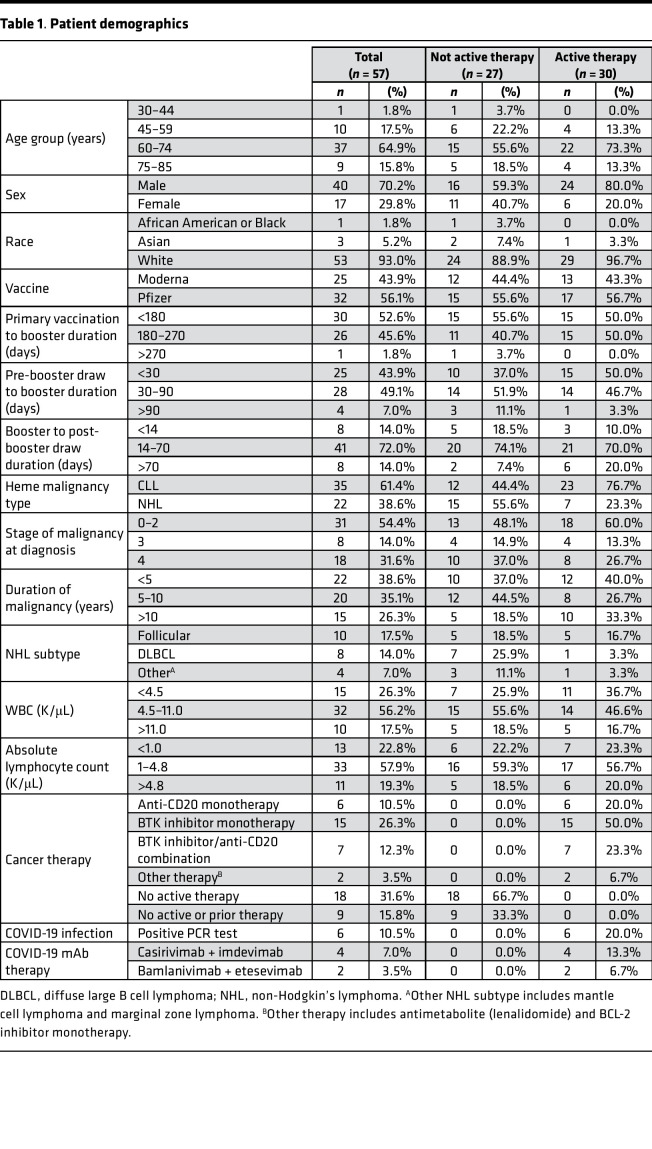
Patient demographics

## Version 1 02/07/2023

In-Press Preview

## Version 2 03/22/2023

Electronic publication
